# Insight into SARS-CoV-2 Omicron variants in Saudi Arabian genomic isolates

**DOI:** 10.15537/smj.2022.43.11.20220381

**Published:** 2022-11

**Authors:** Doaa Bahaa E. Darwish

**Affiliations:** *From the Biology Department, Faculty of Science, University of Tabuk, Tabuk, Kingdom of Saudi Arabia; and from the Botany Department, Faculty of Science, Mansoura University, Mansoura, Egypt.*

**Keywords:** Omicron, mutations, VOC, spike, SARS-CoV-2

## Abstract

**Objectives:**

To investigate the emergent mutations involved in the evolutionary stages of the virus for better management of pandemic.

**Methods:**

This cross-sectional genomic investigation was performed on February 28, 2022, at the Biology Department, Faculty of Science, Tabuk University. Numerous mutations were searched in genomic isolates of Omicron variant prevalent in the Kingdom of Saudi Arabian. Whole-genome sequences were retrieved from genomic databases and were subjected to the Global Initiative on Sharing Avian Influenza Data (GISAID) CoVsurver for the Omicron variant detection and mutations.

**Results:**

Approximately 8.755 million SARS-CoV-2 genomes were reported to GISAID on February 28, 2022, of which 1270 have been reported from the Kingdom of Saudi Arabia. Among the 1270 genomes, 30 were Omicron variants. Among the Saudi Arabian genomes, 30 were detected as Omicron variants. Twenty-four unique mutations have been detected in membrane, envelope, spike and non-structural proteins (NSP) 12, NSP3, and NSP2. Ten of these unique mutations have been detected in spike protein.

**Conclusion:**

The current study provides useful information for further experimental investigation of mutation’s effects on virus transmission, severity, and vaccine efficacy.

**T**he emergence of coronavirus disease 2019 (COVID-19) caused by SARS-CoV-2 (severe acute respiratory syndrome coronavirus 2) cause a major public health issue due to viral evolutionary stages, which have increased transmissibility, and potential immune escape. Different evolutionary stages are continued, and new variants of concern/interest (VOC/I) are emerging. Mutations on the viral spike protein may affect SARS-CoV-2 binding to the human cell surface receptor called ACE2 (angiotensin-converting enzyme 2) and antibodies. These VOCs share some important mutations in which E484K is shared by B.1.351 and P.1 variants, decreasing the binding affinity with neutralizing antibodies.^1-3^

Omicron, a new SARS-CoV-2 VoC, was reported. The Omicron’s first sequenced genome was published in Botswana on November 2021, and has some deletions and more than 30 spike mutations.^4,5^

In the current investigation, we screened 8.755 million genomes in Global Initiative on Sharing Avian Influenza Data (GISAID) for the Omicron variant submitted from Saudi Arabian. A total of 30 genomic isolates were Omicron variants.

## Methods

This cross-sectional study was conducted on February 28, 2022, at the Biology department, Faculty of Science, Tabuk University. The complete genomic sequences were searched in the GISAID (February 28, 2022) public database receiving SARS-CoV-2 genomic data from worldwide.^6^ The database is freely accessible to researchers across the globe. This genomic information has been generated and submitted to GISAID for better public health research activities in over 3,500 research institutions.

Global Initiative on Sharing Avian Influenza Data enables several web tools to perform SARS-CoV-2 variant identification. A total of 8.755 million genomes of SARS-CoV-2 were reported on February 28, 2022, to the GISAID server. The variants were screened using the variants options of GISAID.

Among the 8.755 million genomes reported worldwide, Saudi Arabian genomic isolates were screened using the GISAID location option. At the same time, Omicron variants have been searched in the variants option.

The Omicron variant data was retrieved from GISAID in Fasta format and subjected to the CoVsurver (www.gisaid.org). The server aligns the query sequences with Wuhan reference (NC_045512) for variant screening and identification of mutations in all viral proteins. The server also annotates the nucleotide mutations into amino acid changes in all viral proteins.

The inclusion criteria: SARS-COV-2 complete genomic isolates were submitted from the Kingdom of Saudi Arabia to GISAID and NCBI virus databases. Only Omicron variants were included in this study. And the exclusion criteria: SARS-COV-2 incomplete genomic isolates and variants other than Omicron were excluded.

Simple statistics in an excel sheet were executed to identify the unique and most common mutational pattern in all structural and non-structural proteins of Omicron variants in Saudi Arabian isolates. Locations of each unique and reported mutation were recorded.

Ethical approval from the Institutional Ethical Board, The University of Tabuk, Saudi Aarabia is not required because the genomic data were retrieved from public databases and were not linked with individual patients. The data is already available in the GISAID public database. Moreover, the study did not involve collecting data directly from humans; neither have the results been linked to patients.

## Results

On February 28, 2022, approximately 8.755 million SARS-CoV-2 isolates, 1270, were reported from Saudi Arabia, of which 30 were Omicron variants (Supplementary file S1). A total of 1651 mutations had been detected, among which 24 were unique to GISAID. These unique mutations have been detected in membrane (M), spike (S), envelope (E), and non-structural proteins (NSP) 12, NSP3, and NSP2. Among the 24 unique mutations, ten have been detected in S protein (Table 1).

**Table 1:** - List of unique mutations in ten genomic isolates.

Genomic isolate	Unique mutations
hCoV-19/Saudi Arabia/KFSHRC_0103Y/2021|EPI_ISL_8979486|2021-12-18	(NSP3_N1220>L, NSP3_E1213>L, M_C33>W)
hCoV-19/Saudi Arabia/KFSHRC_0105Y/2021|EPI_ISL_8980284|2021-12-17	(N_ins67P)
hCoV-19/Saudi Arabia/KFSHRC_0101I/2021|EPI_ISL_9034121|2021-12-21	(NSP5_D187>C)
hCoV-19/Saudi Arabia/KFSHRC_0103I/2021|EPI_ISL_9034444|2021-12-20	(Spike_Q506>L, Spike_Y505>W, Spike_P507>E)
hCoV-19/Saudi Arabia/KFSHRC_0104I/2021|EPI_ISL_9034534|2021-12-21	(Spike_L806>I, E_L39>Y, E_Y42>S)
hCoV-19/Saudi Arabia/KFSHRC_0107I/2021|EPI_ISL_9034549|2021-12-21	(Spike_A93>L)
hCoV-19/Saudi Arabia/KFSHRC_0117Y/2021|EPI_ISL_9034733|2021-12-20	(Spike_Q779>T, Spike_V781>K)
hCoV-19/Saudi Arabia/KFSHRC_0118Y/2021|EPI_ISL_9034734|2021-12-20	(NSP2_I613>F, NSP2_L611>T, NSP2_M609>V, NSP5_G109V, NSP12_A34stop, NSP12_I37>L, NSP12_D36>V, Spike_K795Y)
hCoV-19/Saudi Arabia/KFSHRC_0119Y/2021|EPI_ISL_9034789|2021-12-19	(Spike_N343>Y)
hCoV-19/Saudi Arabia/KFSHRC_0130Y/2021|EPI_ISL_9089309|2021-12-20	(Spike_P139>L)

**Figure 1:**
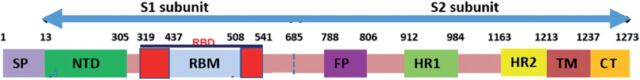
- Domain organization of spike protein. NTD: N-terminal domain, RBD: receptor binding domain, RBM: receptor binding motif, FP: fusion peptide, HR: Heptapeptide repeat, TM: transmembrane, CT: cytoplasmic tail

Four of the spike’s unique mutations (Spike_Q506>L, Spike_Y505>W, Spike_P507>E, Spike_N343>Y) were found in the RBD region (Figure 1), which is involved in binding affinity with the human ACE2 receptor. Two have been detected in NTD (Spike_A93L, P139L) of the spike S1 subunit and Spike_L806>I, Spike_Q779>T, Spike_V781>K, and Spike_K795>Y in the S2 subunit (Figure 1). Among the structural protein mutations, 2 unique mutations (E_L39>Y, E_Y42>S) in E proteins and one in M (M_C33>W) were also detected. One insertion was also detected in N proteins at position 67 (ins67P).

Mutations unique to GISAID were also detected in NSPs. These are in NSP3 (NSP3_N1220>L, NSP3_E1213>L), NSP5 (NSP5_D187>C, NSP5_G109>V), and NSP12 (NSP12_A34stop, NSP12_I37>L, NSP12_D36>V). A single genomic isolate has also detected 3 unique mutations in NSP2 (I613>F, L611>T, and M609>V). Among the NSP12 mutations, one was non-sense (NSP12_A34stop), and 2 were non-synonymous mutations (NSP12_I37>L and NSP12_D36>V). One unique mutation in SARS-CoV2 M proteins (C33>W) and 2 were also detected in E proteins (L39>Y and Y42>S).

## Discussion

Omicron harbors different types of mutations in RBD of S protein which may impact adaptation and strengthen the ACE2-RBD binding.^7-9^ Different VOCs have some specific mutations in the RBD of spike protein.^1,2^

The Kingdom of Saudi Arabia (KSA) has taken effective measures to prevent the spread of SARS-CoV-2. The KSA has effective screening to protect the public, and genomic sequencing is also being performed on the population for virus detection. Novel variants with increased transmission and pathogenicity have been reported worldwide. In the current study, we screened whole genome sequences of SARS-CoV-2 submitted from KSA in GISAID to screen mutations in virus spikes and other target proteins for better understanding the virus evolutionary stages circulating in KSA.

Several precautions are needed for better management of the COVID19 Omicron variant. Maximum genomic sequencing and sharing these sequences with public databases for mutation rate and pattern and their proper assessments to observe the impacts on vaccines efficacy, diagnosis, or social precautions. Vaccine efficacy was slightly lower in earlier VOC; however, the potency of the BNT162b2 vaccine was the same against the beta variant.^10^

Omicron variants harbor some unique mutations (supplementary files S1), among which 4 of spike’s unique mutations (Spike_Q506L, Spike_Y505W, Spike_P507E, Spike_N343Y) were found in the RBD region. These RBD region mutations might impact virus transmission and disease severity. The earliest Omicron detected in South Africa has more than 30 mutations in S proteins which are known for increased infectivity, transmissibility, and immune escape ability.^5^

However, the potency of vaccines was good against the previous variants. Approximately 90%, of vaccine efficacy has been reported from Qatar against delta-variant; however, data regarding Omicron and vaccine efficacy is limited.^11^ Some data in New York exhibit different levels of efficacies for different vaccines. However, mutations reported in the RBD region need to be investigated for virus binding affinity with human ACE2 and the virus transmission rate in the population. A recent study on the 2 doses of Pfizer–BioNTech messenger ribonucleic acid (mRNA) vaccine (BNT162b2) in South Africa found that vaccine effectiveness was 70% with a 95% confidence interval.^12^ A recent study analyzed 886,774 infected persons with the Omicron and 204,154 infected with the delta variant for vaccine efficacy.^13^ For all primary course and booster vaccine combinations, vaccine effectiveness was higher for the SARS-CoV-2 delta variant than the Omicron. A primary course of BNT162b2 followed by the booster of mRNA-1273 enhanced the vaccine’s effectiveness (73.9%) at 2 to 4 weeks. Further investigations are needed to find the geographic-specific mutations in Omicron variants and their effect on vaccine efficacy.

### Study limitation

This study only analyzed SARS-CoV-2 Omicron variant genomic isolates from the Kingdom of Saudi Arabia. A broader range of genomic isolates of different geographical locations may provide a better understanding.

In conclusion, the Omicron variant harbored some unique mutations in Saudi Arabian isolates where Spike_Q506>L, Spike_Y505>W, Spike_P507>E, and Spike_N343>Y were present in the spike RBD domain that may affect virus transmissibility. One unique mutation in SARS-CoV2 M and two were also detected in E protein. Among the NSP mutations, NSP3, NSP3, NSP5, and NSP12 also harbored some unique mutations. Further studies are needed to explore whether these mutations play any role in the Omicron immune escape capability. Continuous molecular epidemiology is required for further analysis of virus evolutionary stages in Saudi Arabia.
